# Chronic exposure to cadmium induces a malignant transformation of benign prostate epithelial cells

**DOI:** 10.1038/s41389-020-0202-7

**Published:** 2020-02-17

**Authors:** Balaji Chandrasekaran, Nisha R. Dahiya, Ashish Tyagi, Venkatesh Kolluru, Uttara Saran, Becca V. Baby, J. Christopher States, Ahmed Q. Haddad, Murali K. Ankem, Chendil Damodaran

**Affiliations:** 1 Department of Urology, University of Louisville, Louisville, KY USA; 2 Pharmacology and Toxicology, University of Louisville, Louisville, KY USA

**Keywords:** Oncogenes, Prostate cancer, Oncogenes, Prostate cancer

## Abstract

Epidemiological evidence suggests that cadmium (Cd) is one of the causative factors of prostate cancer, but the effect of Cd on benign prostatic hyperplasia (BPH) remains unclear. This study aimed to determine whether Cd exposure could malignantly transform BPH1 cells and, if so, to dissect the mechanism of action. We deciphered the molecular signaling responsible for BPH1 transformation via RNA-sequencing and determined that Cd induced the expression of zinc finger of the cerebellum 2 (ZIC2) in BPH1 cells. We noted Cd exposure increased ZIC2 expression in the Cd-transformed BPH1 cells that in turn promoted anchorage-independent spheroids and increased expression of stem cell drivers, indicating their role in stem cell renewal. Subsequent silencing of ZIC2 expression in transformed cells inhibited spheroid formation, stem cell marker expression, and tumor growth in nude mice. At the molecular level, ZIC2 interacts with the glioma-associated oncogene family (GLI) zinc finger 1 (GLI1), which activates prosurvival factors (nuclear factor NFκB, B-cell lymphoma-2 (Bcl2), as well as an X-linked inhibitor of apoptosis protein (XIAP)) signaling in Cd-exposed BPH1 cells. Conversely, overexpression of ZIC2 in BPH1 cells caused spheroid formation confirming the oncogenic function of ZIC2. ZIC2 activation and GLI1 signaling induction by Cd exposure in primary BPH cells confirmed the clinical significance of this oncogenic function. Finally, human BPH specimens had increased ZIC2 versus adjacent healthy tissues. Thus, we report direct evidence that Cd exposure induces malignant transformation of BPH via activation of ZIC2 and GLI1 signaling.

## Introduction

Benign prostatic hyperplasia (BPH) is a chronic urological disorder characterized by noncancerous enlargement of the prostate gland^1,2^. Both BPH and prostate cancer (CaP) share similar etiology and pathophysiological factors^3–5^. Although, BPH to CaP conversion is controversial, the published evidence suggests that BPH patients have a two to threefold increased risk for CaP and a two to eightfold increased risk for CaP-associated mortality^6^. More recently, a comprehensive review reported an association between BPH and CaP, suggesting that BPH is a risk factor for CaP^7^. While the possibility exists that BPH patients have an increased risk of developing CaP and CaP-associated mortality, it remains unclear how and why BPH patients are at risk for developing CaP.

Cadmium (Cd) is a known metal carcinogen and is one of the most abundant occupational and environmental pollutants found in air, soil, water, dietary products, and tobacco smoke^8^. Epidemiological studies have reported Cd could be a potent prostate carcinogen because it is found in significantly higher levels in tissues and plasma of CaP patients than healthy controls^9^. Cd is an endocrine disruptor in experimental models^10^, supporting the hypothesis that this metal carcinogen can potentially induce the development of hormone-dependent tumors in humans, including that of the breast and uterus^11,12^. However, its molecular effects on malignant transformation of BPH cells remain elusive.

Deregulation of transcription factors, such as the zinc finger of cerebellum 2 (ZIC2) has been linked to heavy metal exposure and metal-induced carcinogenesis^13^. ZIC2 plays a regulatory function in pluripotent and self-renewal of cancer stem cells (CSCs)^14,15^ and is also highly expressed and involved in tumorigenesis of various cancer types, including CaP^14–16^. As a zinc finger transcriptional factor, ZIC2 binds with the zinc finger domains of other protein families, including glioma-associated oncogene (GLI) in either a synergistic or antagonistic manner^17,18^. GLI proteins are also the downstream targets of the Sonic hedgehog (Shh) pathway that are an important therapeutic target for CaP treatment. Several studies have demonstrated growth arrest in CaP cell lines and xenografted tumors in mice^19,20^ following the inhibition of Shh signaling. Shh-associated secretory protein binds and inactivates patched1 (PTCH1), resulting in the release of smoothened protein and activation of GLI1, GLI2, and GLI3 receptors. GLI1 predominantly functions as a transcription activator, while GLI2 and GLI3 function as either an activator or repressor^21^. GLI1 activation initiates the expression of downstream target genes involved in proliferation (cyclin D1), survival (B-cell lymphoma-2, Bcl2), metastasis (Snail), and stem cell activation (Nanog and SOX2)^22^. Activated GLI1 and GLI2 proteins can also directly promote the expression of a group of genes involved in the process of epithelial mesenchymal transition (EMT)^23^.

Here, for the first time, we report direct evidence that Cd exposure induces malignant transformation of BPH1 cells that exhibit an aggressive phenotype similar to that of CaP. In addition, our results suggest that chronic exposure of Cd to BPH1 cells was responsible for stem cell renewal, proliferation, and tumorigenesis of the transformed cells.

## Materials and methods

### Cell lines and reagents

The BPH1 cell line was a kind gift from Dr. Simon W. Hayward (Northshore Research institute University of Chicago, Pritzker School of Medicine). The cells were authenticated by Genetical Cell Line Testing (Burligton, NC, USA). The cells were treated with 10 µM Cd (Sigma, Dallas, TX, USA) for 1 year and transformed into a malignant phenotype. The transformed cells were named Cd-transformed BPH1 (CTBPH1). Human normal prostate epithelial cells (RWPE-1) were purchased from the American Type Culture Collection (Manassas, VA, USA). BPH1 and CTBPH1 cells were cultured in RPMI medium supplemented with 10% fetal bovine serum, and 1% antibiotic and antimycotic solution in a humidified atmosphere of 5% CO_2_ at 37 °C in an incubator. The RWPE-1 cells were cultured in keratinocyte serum-free medium containing l-glutamine, Epidermal Growth Factor (EGF) and bovine pituitary extract (BPE).

### Cell viability assays

Cell viability assays were performed using the trypan blue exclusion method or the 3-(4, 5-dimethylthiazol-2-yl)-2, 5-diphenyltetrazolium-bromide (MTT) assay as described previously^24^. BPH1 cells following exposure to 10 µM Cd for 24, 48, and 72 h.

### Soft agar colony formation assay

Colony formation assays were performed on Cd-exposed BPH1 and CTBPH1 cells to monitor anchorage-independent growth via the CytoSelectTM 96-well in vitro tumor sensitivity assay kit (Cell Biolabs, Inc., San Diego, CA, USA)^25^. A group of at least 50 or more cells were considered as a colony and those colonies were counted.

### Migration and invasion assays

Invasion assays were performed on BPH1, 6-month Cd-exposed BPH1, CTBPH1 (12-month Cd-transformed cells), ZIC2^+^, and ZIC2^−^ using Boyden chambers equipped with polyethylene terephthalate membranes with 8-μm pores (BD Biosciences) as described previously^26^. Similarly, migration assays were performed for Cd-exposed BPH1 cells in six-well plates as described previously^24^.

### Sphere formation assay

Spheroids of ZIC2^+^ and ZIC2^−^ cells were generated by forced floating method using 96-well round-bottom Ultra Low Attachment plates (Corning®, New York, USA). Single-cell suspension of ZIC2^+^ and ZIC2^−^ cells at a density of 2 × 10^3^ cells in 200 μl of respective culture media supplemented with Matrigel^TM^ (354254, Corning) was loaded into each well. Then, the morphology of the spheroids, cell growth were characterized over a 7-day culture period in triplicate.

### Overexpression and siRNA transfection

BPH1 cells were seeded in six-well plates at a density of 3 × 10^5^ cells/well. After 24 h, the cells were transiently transfected with short-interfering RNA (siRNA) specific for ZIC2 or GLI1, or control siRNA, or an overexpression plasmid specific for ZIC2 for 48 h. Lipofectamine® 2000 was used as a transfection reagent. After 48 h, lysates were prepared and western blotting was performed.

### Immunoprecipitation (IP) and western blot

BPH1, transforming cells (2, 4, 6, 8, 10, 12, 14, and 16 months), and CTBPH1 cells were seeded in six-well plates and incubated for 24 h. Cell lysates were prepared, and western blotting was performed using specific antibodies against ZIC2 (ab150404), GLI1 (ab151796), Shh (ab53281), NANOG (cell signaling #3608), ZIC2 immunohistochemistry (Sigma AU35821), CD44 (cell signaling #5604), Poly(ADP-ribose) polymerase (PARP) (Cell signaling #9542), NFκB-p65 (Cell signaling #8242), Bcl2 (Cell signaling #2872), Bcl_xL_ (Santa curtz 7B2.5), X-linked inhibitor of apoptosis protein (XIAP; Cell signaling #2045), cleaved caspase-3 (Novus Biologicals NB100-56112), PTCH1 (ab53281), NOTCH1 (Cell signaling #3608), SOX2 (Cell signaling #3579), E-cadherin (Cell signaling #3195), N-cadherin (Cell signaling #13116), Slug (Cell signaling #9585), and β-catenin (Cell signaling #8480). Protein–antibody complexes were visualized using enhanced chemiluminescence as previously described^25^. For immunoprecipitation experiments, the protein samples were immunoprecipitated with ZIC2 antibody at 4 °C under agitation overnight following protein extraction using radioimmunoprecipitation assay (RIPA) buffer; the immunoprecipitated protein was then pulled down with protein A-agarose beads (Thermo Fisher Scientific, Rockford, IL) at 4 °C under rotary agitation for 3 h. Immunoprecipitates were washed three times with RIPA buffer. After centrifugation, the pellets were resuspended in sample buffer and heated for 5 min at 95 °C for sodium dodecyl sulfate–polyacrylamide gel electrophoresis followed by immunoblot analysis.

### Real-time Quantitative PCR (RT-qPCR)

Total RNA was extracted from BPH1, Cd-exposed BPH1 (24, 48 and 72 h), CTBPH1, and ZIC2 siRNA-treated CTBPH1 cells and RT-qPCR performed for ZIC2, SOX2, Nanog, CD44, and Notch1 expressions as described previously^27^.

### Immunofluorescence and immunohistochemistry analysis

Immunofluorescence for ZIC2 and GLI1 expression was performed on Cd-exposed BPH1 and CTBPH1 cells, as described previously^28^. BPH tissue microarray and xenograft tumor tissues were examined for ZIC2, GLI1, and p65 expression using immunohistochemistry analysis.

### Cell sorting of ZIC2^+^ and ZIC2^−^ cells

Cd-exposed BPH1 or CTBPH1 (1 × 10^7^) cells were incubated with 2.0 μl of fluorochrome-conjugated ZIC2 antibody before being sorted using fluorescence-activated cell sorting (FACS). Cells were analyzed on a Cytopeia Influx FACS using Spigot. Cell subpopulations were separated based on the surface antibody labeling and collected by discriminatory gating. After selecting for ZIC2^+^, cell suspensions were sorted from BPH cell population.

### Xenograft studies

Five-week-old male BALB/c (nu/nu) athymic mice were purchased from The Jackson Laboratory and housed in the University of Louisville vivarium under pathogen-free conditions. All experimental animals were maintained in accordance with the Institutional Animal Care and Use Committee approval and approval was obtained from the ethical committee of the University of Louisville, KY. The mice were randomly divided into four groups of eight each (a unique identification number was assigned to the animals, the numbers were drawn randomly and animals were logically assigned to different groups). At 7 weeks of age, the mice were subcutaneously injected with BPH1, CTBPH1, ZIC2^+^, and ZIC2^−^ cells (1 × 10^6^) in 50 μl Matrigel (Corning). The mice were monitored twice weekly, and the tumor volumes were measured once per week. At the end of experiments, the mice were euthanized via CO_2_ asphyxiation; the tumors were removed and fixed in 10% formalin for histopathological studies.

### Statistical analysis

All statistical analysis used GraphPad Prism 8.0a software (GraphPad Software, Inc., La Jolla, CA). An unpaired two-tailed Student’s *t*-test was performed for two-group comparisons and one-way analysis of variance analysis was performed for multiple group comparisons. The statistical significance was set at a *P* < 0.05, and values were presented as means ± SD.

## Results

### Acute Cd exposure promotes Shh/ZIC2 expression in BPH1 cells

**Fig. 1:**
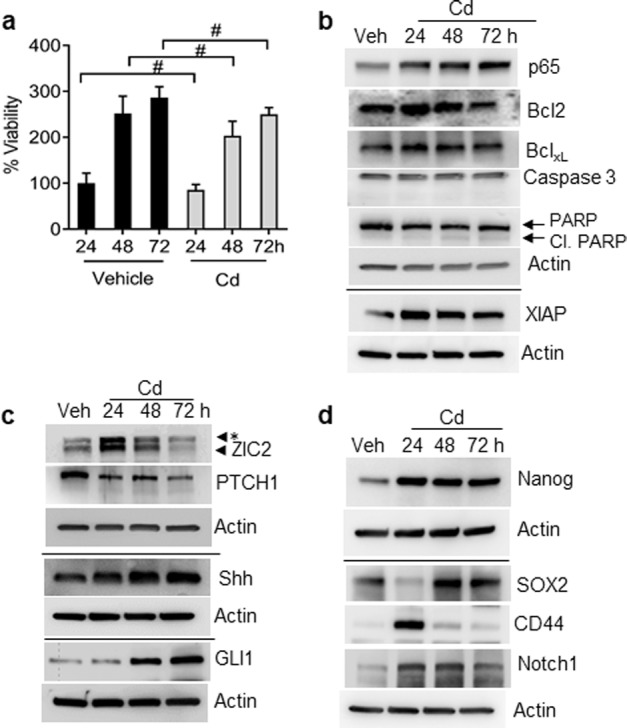
Effect of acute Cd exposure on BPH1 cell proliferation, Shh signaling activation, and stem cell markers in a time-dependent manner. **a** BPH1 cells were exposed to Cd for 24, 48, and 72 h; cell proliferation was determined by MTT assay. **b** Cells were incubated with or without 10 µM Cd for the indicated time. Cell lysates were subjected to western blotting using antibodies against p65, Bcl2, Bcl_xL_, XIAP, cleaved caspase-3, cleaved PARP, and β-actin was used as loading control. **c** Total cell lysates extracted from cells exposed to Cd (24, 48, and 72 h) were subjected to western blot analysis using specific antibodies against Shh signaling. **d** Cell lysates from cells exposed to Cd (24, 48, and 72 h) were used to determine the protein level of Nanog, SOX2, CD44, and Notch1 by western blot analysis. β-actin was used as the loading control. #, Not significant; *, nonspecific band.

We investigated whether acute Cd exposure impacted BPH1 cell viability. Although, growth inhibition of the Cd-exposed BPH1 cells was seen, this result was not statistically significant versus vehicle-treated cells (Fig. 1a). Subsequent examination of prosurvival and proapoptotic proteins using western blots revealed that Cd exposure increased the expression of survival markers, such as p65, Bcl2, Bcl_XL_, and XIAP but did not significantly alter the expression of proapoptotic proteins cleaved caspase-3 and PARP (Fig. 1b).

Next, a global RNA-sequence analysis was performed to determine the molecular events in Cd-exposed BPH1 cells. The results indicated that the induction of ZIC2 and Shh signaling appeared to be a prominent event in the transformation of Cd-exposed BPH cells (data not shown). Western blots and qRT-PCR confirmed that ZIC2 and Shh pathway markers were upregulated, validating our transcriptomic analysis. The results confirmed the activation of Shh signaling in Cd-exposed BPH1 cells, as determined by a decrease in PTCH1 expression and corresponding upregulation of Shh, GLI1, and ZIC2 (Fig. 1c). While Cd exposure induced the expression of GLI1, no significant alterations were noted in expressions of other GLI family members (GLI2 and GLI3) in the BPH1 cells (data not shown). Stem cell renewal is a function of ZIC2 and further confirms its activation following Cd exposure; thus, we also examined the expression of stem cell markers in Cd-exposed BPH1 cells. The results showed increased expression of Nanog, SOX2, CD44, and Notch1 confirming that ZIC2 activation may be a key response to Cd exposure in BPH1 cells (Fig. 1d).

**Fig. 2:**
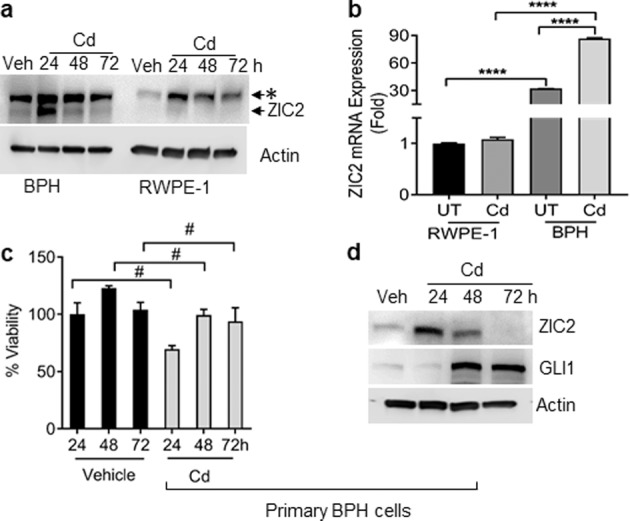
Acute exposure of Cd induces ZIC2 in primary human BPH cells and BPH1 cells. **a** Cells were incubated with 10 µM Cd for the indicated time periods. Western blot analyses comparing ZIC2 expression in BPH1 cells (left four lanes) and RWPE-1 (right four lanes). **b** mRNA expression of ZIC2 in Cd-treated RWPE-1 and BPH1 cells. **c** Primary human BPH cells were exposed to Cd for indicated time points, MTT assay was performed to analyze the effect on cell viability. **d** Cell lysates from primary human BPH cells exposed to Cd (24, 48, and 72 h) were used for western blot analysis using specific antibodies against ZIC2 and GLI1. β-actin was used as the loading control. ****p < 0.0001 and #, not significant; *, nonspecific band.

To further confirm that this activation is specific to Cd-exposed BPH1 cells, we assessed ZIC2 levels in RWPE-1 cells following acute Cd exposure. No changes in ZIC2 mRNA and protein expression were noted in Cd-exposed RWPE-1 cells (Fig. 2a, b), suggesting that Cd activation of ZIC2 was specific to BPH1 cells.

Although the use of immortalized cell lines is imperative for metal carcinogenesis especially for transformation assay, the effect of the immortalization process itself on the molecular signature of the metal carcinogen-induced transformation remains a concern for researchers. To address this, we compared effect on cell viability and ZIC2/GLI1 expression on primary human BPH cells with normal primary BPH cells following Cd exposure. The Cd exposure reduced the cell viability (not significantly) as seen in primary human BPH cells and increased the expression of ZIC2 and GLI1 (Fig. 2c, d). This confirmed that the molecular signature of Cd is not compromised in immortalized BPH1 cells.

### Chronic exposure to Cd induces malignant transformation of BPH1 cells

**Fig. 3:**
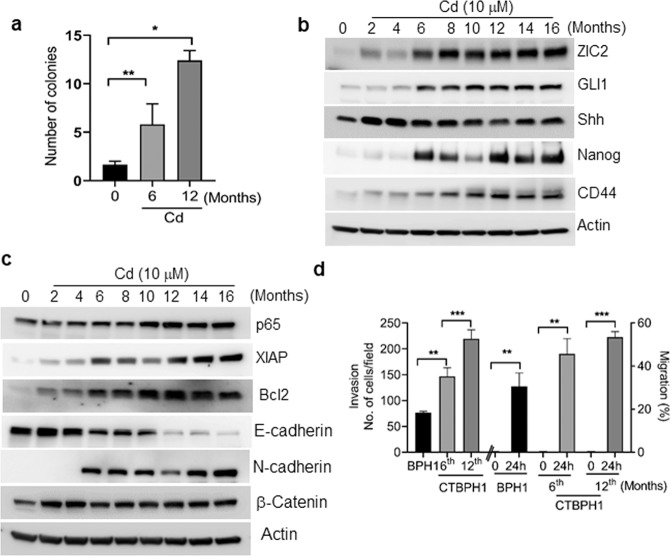
Effect of chronic exposure to Cd on soft agar assay, shh signaling, stem cell properties, cell survival, and EMT marker’s expression in CTBPH1 cells. **a** Soft agar assay for colony formation in Cd-treated BPH1 cells after 6 and 12 months. **b** Total cell lysate extracted from BPH1 cells exposed to Cd (10 µM) up to 16 months was used to immunoblot ZIC2, GLI1, Shh, Nanog, and CD44, **c** p65, XIAP, Bcl2, EMT markers, and β-actin was used as a loading control. **d** A migration assay and Boyden chamber invasion assay were used to assess cell migration and invasion ability of CTBPH1 cells. **p < 0.01 and ***p < 0.001.

In order to develop an in vitro model of Cd-induced malignant transformation, we chronically exposed BPH1 cells to 10 μM Cd for up to 16 months. We periodically performed anchorage-independent growth assays to determine the malignant transformation of Cd-exposed cells. BPH1 cells began forming colonies after 6 months of Cd exposure, and the number of the colonies increased with exposure time. This confirmed that Cd exposure induced the malignant transformation of BPH1 cells (Fig. 3a).

We next performed western blot analysis to confirm whether the molecular signature of chronic Cd exposure was similar to acute exposure in the BPH1 cells. The results showed that similar to acute exposure, chronic Cd exposure also increased the expression of ZIC2, Shh, and GLI1. Upregulation of prosurvival proteins p65, XIAP, and Bcl2 stem cell markers Nanog and CD44 was also observed (Fig. 3b, c). Interestingly, we observed a time-dependent change in the expression of EMT markers and β-catenin in the chronic Cd-exposed BPH1 cells with the upregulation of N-cadherin, β-catenin, and downregulation of E-cadherin (Fig. 3c). To corroborate the western blot results further, we also performed cell invasion and migration assays in Cd-transformed cells. The results demonstrated that chronic Cd exposure significantly increased the invasive and migratory properties of cells exposed cells for 6 and 12 months versus vehicle-treated control cells (Fig. 3d). These results collectively indicate that Cd exposure significantly induces the invasion and migration abilities of transformed cells.

Although, the BPH1 cells were chronically exposed to Cd up to 16 months to induce malignant transformation, we did not observe any significant phenotypic changes in the cells exposed beyond 12 months. Thus, we considered the cells exposed to Cd for 12 months to be completely transformed and will refer to them as CTBPH1 below.

### ZIC2 promotes CSC characteristics and EMT phenotype in malignant transformed BPH1 cells

**Fig. 4:**
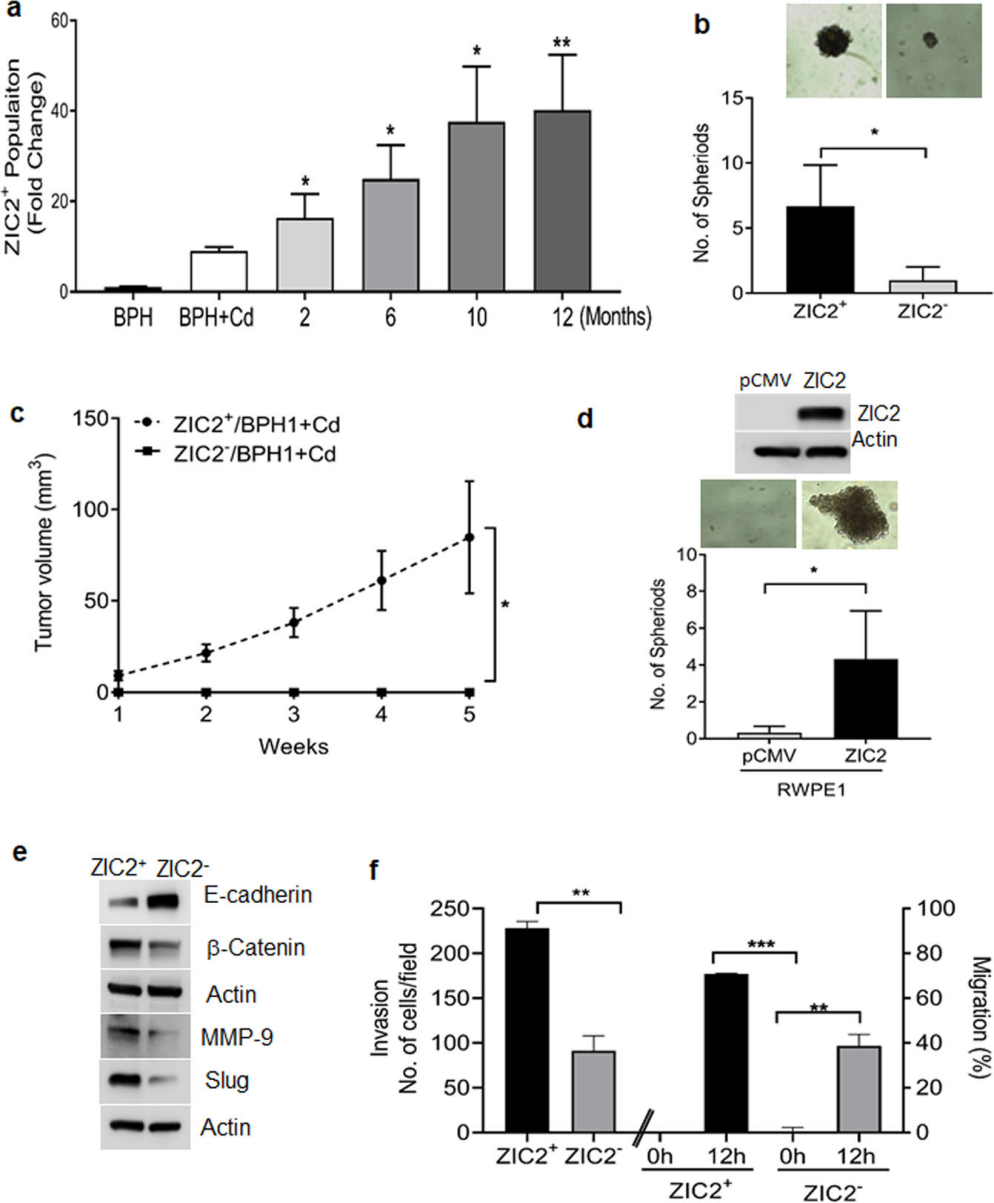
ZIC2^+^ cancer cells have properties of CSCs. **a** Fluorescence-activated cell sorting (FACS) analysis of BPH1 cells exposed to 10 µM Cd (0, 2, 6, 10, and 12 months) expressing ZIC2. **b** Spheroid formation assay for ZIC2^+^ and ZIC2^−^ cell population harvested from FACS. **c** Tumor burden in mice xenotransplanted with ZIC2^+^ and ZIC2^−^ (cells furthest from ZIC2^+^ gating) was analyzed during 5 weeks of study. **d** Spheroid formation assay for RWPE1 cells overexpressing ZIC2. **e** Cell lysates from ZIC2^+^ and ZIC2^−^ were subjected to western blotting using antibodies against E-Cadherin, β-catenin, slug, and MMP 9. β-actin was used as the loading control. **f** Migration assay and Boyden chamber invasion assay were used to assess cell migration and invasion ability of ZIC2^+^ and ZIC2^−^ cells. **p < 0.01 and ***p < 0.001.

Self-renewal, higher tumorigenicity, sphere formation, and metastatic ability are characteristic properties of CSCs^29,30^. We used FACS to sort the ZIC2-negative and -positive cell populations at specific time points (0, 2, 6, 10, and 12 months) to determine whether Cd-mediated ZIC2 CSCs properties in the transformed BPH1 cells. A gradual increase in the percentage of ZIC2^+^ cells was observed in the transforming cells; the 12-month timepoint had the highest fraction of positive cells (45–50%; Fig. 4a). Interestingly, subsequent spheroid assays demonstrated that only ZIC2^+^ cells formed spheres while ZIC2^−^ cells did not form spheroids (Fig. 4b), confirming that ZIC2 expression was associated with the self-renewal properties of Cd-exposed cells. This was corroborated via xenotransplantation experiments that showed only nude mice injected with Cd-exposed ZIC2^+^ developed tumors versus mice injected with ZIC2^−^ cells (Fig. 4c). To further confirm that ZIC2 is a stem cell driver, we performed sphere formation assay in ZIC2 overexpressed in normal epithelial cells (RWPE-1). As seen in (Fig. 4d) ZIC2 overexpression in normal prostate epithelial cells formed spheroids and no spheroids were seen in control vector transfected cells. We next evaluated the expression of epithelial (E‐cadherin), mesenchymal (β-catenin), in ZIC2^+^ and ZIC^−^ CTBPH1 cells. Increased, MMP 9, slug and β-catenin expression followed by downregulation of E‐cadherin was noted in the ZIC^+^ cells as compared with ZIC^−^ CTBPH1 cells. (Fig. 4e).

Furthermore, invasion and migration assays demonstrated that ZIC2^+^ cells showed increased invasive and migratory properties (Fig. 4f) versus ZIC2^−^ cells. These results suggest that ZIC2 may aid in the transformation of Cd-exposed BPH1 by inducing mesenchymal phenotype and promoting stem cell renewal.

### Silencing ZIC2 abolishes self-renewal potency of CTBPH1 cells

**Fig. 5:**
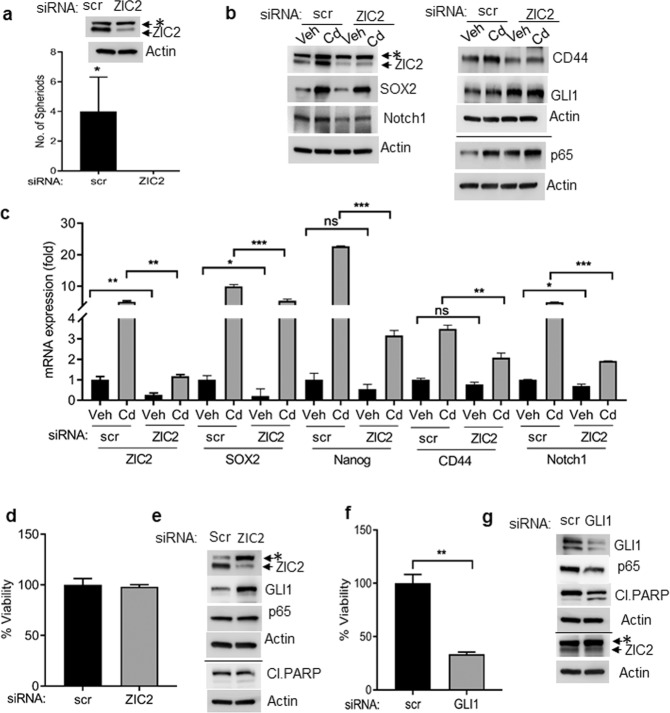
Effect of ZIC2 and GLI1 siRNA knock down on viability, survival, and stem cell markers expression in CTBPH1 cells. **a** Spheroid formation assay for CTBPH1 cells transfected with scrambled (scr) and ZIC2 siRNA. **b** CTBPH1 cells transfected with scr or ZIC2 siRNA were incubated with or without Cd for 24 h. Cell lysates were subjected to western blotting using antibodies against ZIC2, GLI1, SOX2, CD44, p65, Notch1, and β-actin. **c** RNA isolated from CTBPH1 cells transfected with scr or ZIC2 siRNA and incubated with or without Cd for 24 h was subjected to qRT-PCR analysis using ZIC2, SOX2, Nanog, CD44, and Notch1 primers. **d** Cell viability of CTBPH1 cells treated with scr or ZIC2 siRNA was determined by trypan blue assay. **e** ZIC2, GLI1, p65, and cleaved PARP expression in cell lysates prepared from scr and ZIC2 siRNA-treated CTBPH1 cells. **f** Cell viability of CTBPH1 treated with scr or GLI1 siRNA: The cell viability was determined by trypan blue assay. **g** Cell lysates were prepared from scr and GLI1 siRNA-treated CTBPH1 cells to determine the protein levels of ZIC2, GLI1, p65, and cleaved PARP by western blot analysis. #, not significant, *p < 0.05, **p < 0.01, and ***p < 0.001; *, nonspecific band.

Considering that Cd-mediated ZIC2 activation promoted the self-renewal properties of the CTBPH1 cells, we inhibited the ZIC2 expression in these cells to determine if this altered their self-renewal potency. Spheroid assays showed that silencing ZIC2 expression in CTBPH1 cells completely abrogated the formation of spheroids versus cells transfected with scrambled vectors (Fig. 5a). Subsequent western blots and RT-qPCR assays revealed that silencing ZIC2 expression caused the inhibition of stem markers SOX2, Notch1, and CD44 expression, suggesting that ZIC2 may be a master regulator of stem cell markers (Fig. 5b, c). Interestingly, cell viability assays showed that inhibition of ZIC2 did not inhibit the growth of CTBPH1 cells (Fig. 5d) nor alter the expression of prosurvival (p65) or proapoptotic (PARP cleavage) markers. Additionally, inhibition of ZIC2 failed to alter the levels of GLI1 levels in the CTBPH1 cells (Fig. 5e), suggesting that ZIC2 activation is necessary for stem cell renewal but is not involved in the prosurvival or proapoptotic signaling during Cd-mediated transformation.

### GLI1 knock down decreases the proliferation of CTBPH1 cells

As shown above, Cd exposure induced ZIC2- and Shh-mediated GLI1 signaling in the CTBPH1 cells. We postulated that the induction of prosurvival or inhibition of proapoptotic signaling may be regulated by activation of GLI1. Interestingly, cell viability assays showed that inhibition of GLI1 inhibited the growth of CTBPH1 cells (Fig. 5f). To confirm this, we silenced GLI1 expression in CTBPH1 cells resulting in the upregulation of PARP and concurrent inhibition of p65 expression. Moreover, inhibition of GLI1 failed to alter the levels of ZIC2 levels in the CTBPH1 cells (Fig. 5g).

### Determination of the molecular interplay between ZIC2 and GLI1 activation by Cd

**Fig. 6:**
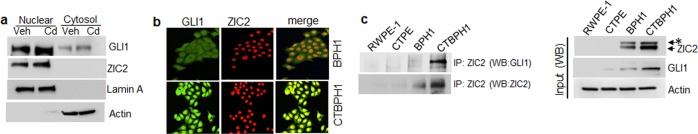
Cd exposure leads to the nuclear translocation and retention of GLI1 and ZIC2. **a** Western blot analysis of cytosolic and nuclear fractions of BPH1 cells treated with 10 µM Cd for 24 h. For loading controls, the same membranes were reprobed with antibodies to β-actin and lamin A for cytoplasmic and nuclear fractions, respectively. **b** Immunofluorescence staining patterns of ZIC2 and GLI1 in BPH1 and CTBPH1 cells. **c** Cell lysates of RWPE-1, CTPE, BPH1 and CTBPH1 cells were immunoprecipitated with anti-ZIC2 antibody. The immunoprecipitates were analyzed by western blot with anti-GLI1; *, nonspecific band.

These results indicate that both ZIC2 and Shh/GLI1 signaling may be necessary for Cd-induced transformation of BPH1 cells. To delineate how the GLI1 and ZIC2 molecularly interact and coordinate their functions during Cd-mediated transformation, we first investigated the localization of these two transcription factors in the CTBPH1 cells. Western blot and immunofluorescence analysis revealed that Cd exposure caused induction and nuclear accumulation of ZIC2 and GLI1 versus vehicle-treated control cells (Fig. 6a, b). Previous studies have suggested that GLI1 physically interacts with ZIC2 protein via its zinc finger domain^31^; thus, we performed IPs to confirm whether Cd-induced BPH carcinogenesis occurs due to the interaction between these two proteins. Unlike the vehicle-treated BPH1 cells that showed a complete absence of GLI1/ZIC2 binding, strong binding was observed in CTBPH1 cells (Fig. 6c). These results suggest that GLI1/ZIC2 interaction plays an important role in the Cd-mediated transformation of BPH1 cells.

### Xenograft analysis of CTBPH1 cells

**Fig. 7:**
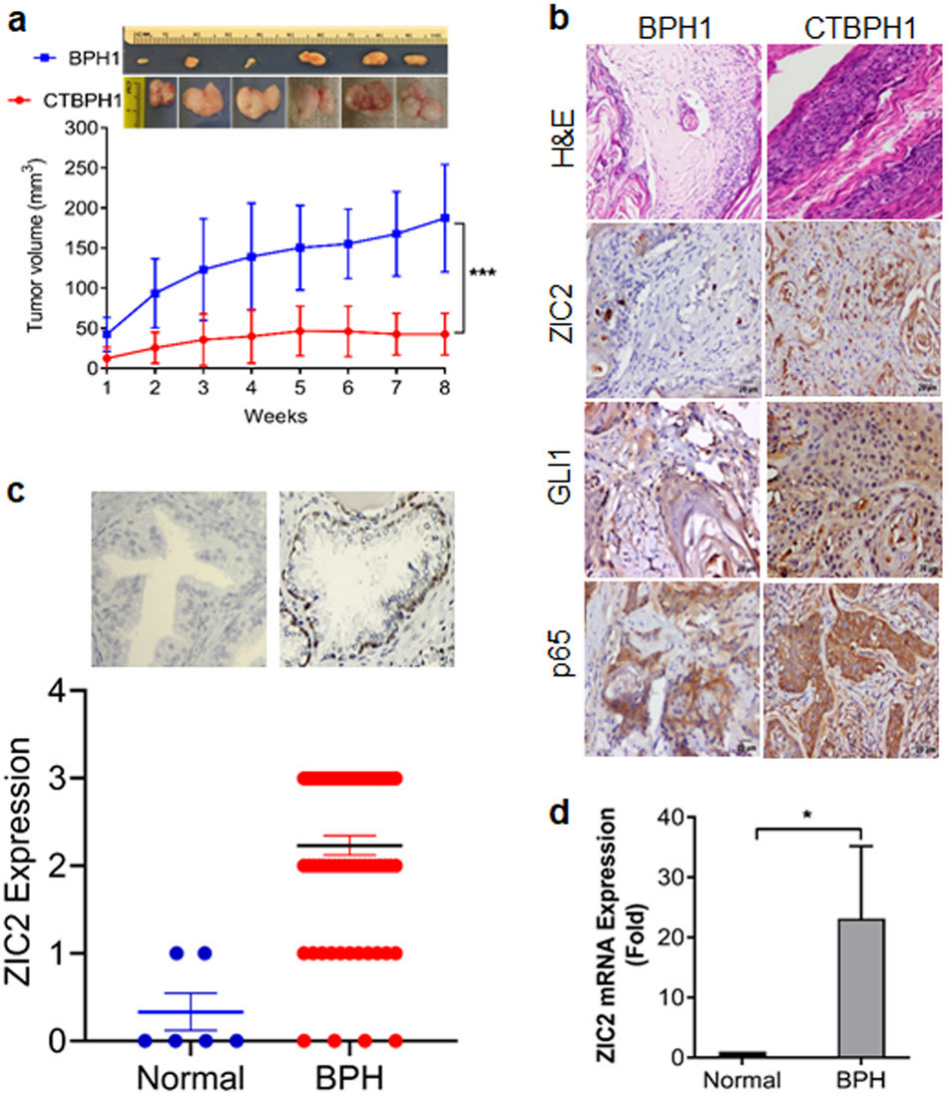
CTBPH1 cells developed tumors in mice. The BPH1 cells exposed to Cd for 12 months were injected subcutaneously into nude mice. After 8 weeks, the mice were euthanized using CO_2_, and the tumor was isolated for further examination. **a** Representative image of excised tumors and graphical representation of the tumor volume in 8 weeks study period. **b** Immunohistochemical analysis of tissue sections derived from BPH1 and CTBPH1 cells for ZIC2, GLI1, and p65. **c** ZIC2 expression in normal human prostate and BPH specimens. **d** mRNA expression of ZIC2 in normal human prostate and BPH human specimens.

Next, we investigated whether CTBPH1 cells could induce tumor formation in nude mice. We observed a progressive increase in tumor growth in mice injected with CTBPH1 cells versus mice injected with vehicle-control cells (Fig. 7a) over a 4-week period post inoculation. Additionally, consistent with the aforementioned in vitro findings, the tumors isolated from the CTBPH1-injected mice demonstrated increased ZIC2, GLI1, and p65 expressions (Fig. 7b).

### ZIC2 expression in human BPH specimens

The immunohistochemistry of BPH tissue microarrays comprising tissue sections from 80 BPH patients showed that ZIC2 immunostaining was predominant in BPH specimens versus normal prostate tissue (Fig. 7c). Examination of the mRNA expression levels of ZIC2 in BPH specimens by qRT-PCR revealed a 20-fold increase in expression in BPH tissue versus normal prostate tissue (Fig. 7d). A higher expression of ZIC2 in BPH specimens suggest that patients with BPH could be susceptible, if exposed Cd for potential malignant transformation.

## Discussion

Although Cd is a well-known human prostatic carcinogen^32^, the underlying mechanisms involved in Cd carcinogenesis remain unclear. Here, we demonstrated that long-term chronic exposure to Cd to BPH1 cells induced transcriptional changes responsible for stem cell renewal, proliferation, and tumorigenesis of the transformed CTBPH1 cells. Cd-mediated ZIC2 activation-initiated stem cell renewal and activation of GLI1: both of these steps promoted cell proliferation of the transformed cells. Thus, we confirmed that the dynamic interaction of ZIC2 and GLI1 are necessary for Cd-induced malignant transformation of BPH cells, a phenomenon not observed in normal prostate epithelial cells.

We previously demonstrated the implantation of Cd-transformed RWPE-1 cells; these cells formed tumors in xenograft models^33,34^. Similarly, this study helped show that chronic exposure to Cd resulted in malignant transformation of BPH1 cells. We previously showed that defective autophagy was responsible for Cd-induced transformation of RWPE-1^33,35^. Here, ZIC2 and GLI1 is responsible for Cd-inducted transformation in BPH1 cells, and it appears that it is specific to BPH1 cells and not to normal prostate epithelial cells.

Earlier studies established the oncogenic role of ZIC2 in pancreatic ductal adenocarcinoma cells by activating the expressions of FGFR3 and ANXA8^36^. Similarly, the stem cell renewal function of ZIC2 was established^37^ by activating the stem cell transcription factors, such as Oct4, Sox2, and Nanog as binding sites^38^. We also found that Nanog and CD44 were upregulated in CTBPH1 cells as compared with vehicle-treated cells. Previous studies demonstrated that CSCs may be responsible for tumor initiation, invasion, distant metastasis, chemo-resistance, self-renewal, and differentiation potential^39^. Thus, we set out to isolate ZIC2^+^ cells from CTBPH1 cells and characterize the ZIC2 function. CSCs are believed to be able to form spheres in culture that can then exhibit extensive similarities to endogenous CSCs in human tumor tissues^40^. Here, sphere formation assays using ZIC2^+^ and ZIC2^−^ cells revealed the increased ability of ZIC2^+^ cell to form spheroids, suggesting that ZIC2 activation imparts CSC properties to Cd-exposed BPH1 cells. Interestingly, upon testing the in vivo potency of ZIC2^+^ versus ZIC2^−^ cells in athymic mice, we observed tumor formation in mice injected with ZIC2^+^ cells but not in those injected with ZIC2^−^ cells. However, our results also demonstrated that the inhibition of ZIC2 expression did alter the expression of cell survival and proapoptotic markers, suggesting activation of an alternative machinery in Cd-exposed cells.

Recently Chan et al.^14^ demonstrated that the overexpression of ZIC2-induced nuclear retention of GLI1: the downstream effector of oncogenic Shh signaling. Other studies have suggested that GLI1 physically interacts with ZIC2 protein via a zinc finger domain^31,41,42^. We found that Cd-induced Shh ligands that in turn inhibited PTCH1 expression leading to the activation of GLI1 and downstream prosurvival targets, such as Bcl2, Bcl_XL_, and XIAP. This strongly suggested that activation of Shh signaling initiated the prosurvival machinery and promotion of cell proliferation in Cd-exposed BPH1 cells; this was later corroborated when we found that silencing GLI1 expression inhibited cell proliferation in CTBPH1 cells. Interestingly, we also noted that the interaction of ZIC2 and GLI1 was stronger in CTBPH1 cells than RWPE-1 and BPH1 cells. This suggests that the oncogenic effects of ZIC2 could be due to the interaction between these two proteins and could in turn aid in the tumorgenicity of the CTBPH1 cells.

Recent studies have demonstrated that Shh activation plays a central role in EMT regulation, involving the loss of cell–cell adhesion, changes in cell morphology, and the propensity to migrate and invade CaP^43,44^. Wei and Shaikh^45^ reported that prolonged Cd treatment in triple-negative breast cancer cells stimulates cell proliferation, adhesion, cytoskeleton reorganization, as well as migration and invasion. Here, we observed that both ZIC^+^ cells and chronic exposure to Cd-induced expression of mesenchymal marker, β-catenin. The Wnt/β-catenin pathway plays a pivotal role in multiple malignancies, including regulating cell proliferation, EMT, and migratory process^46,47^. Moreover, Wnt/β-catenin signaling could also regulate Zic gene expression^48^. Thus, our results strongly suggest that chronic Cd exposure promotes invasion, migration, and EMT in BPH1 cells via activation of the Wnt/β-catenin signaling pathway.

In summary, we found that both exposure of Cd in BPH1 cells causes malignant transformation and tumorigenesis via upregulation of Shh/GLI signaling, as well as ZIC2 activation. We also found that both ZIC2 and GLI1 function as complementary signals initiating stem cell renewal and leads invasion as well as migration, respectively. Thus, they are necessary components for Cd-induced prostate carcinogenesis.
